# MODERATORS OF TREATMENT EFFICACY IN SOCIAL INTELLIGENCE TRAINING WITH CUSTODIAL GRANDMOTHERS

**DOI:** 10.1093/geroni/igae098.0189

**Published:** 2024-12-31

**Authors:** Gregory Smith, Megan Dolbin-MacNab, Frank Infurna, Luxin Hu

**Affiliations:** Kent State University, Kent, Ohio, United States; Virginia Tech, Blacksburg, Virginia, United States; Arizona State University, Tempe, Arizona, United States; Kent State University, Kent, Ohio, United States

## Abstract

In a previously reported randomized clinical trial (RCT), we found that online social intelligence training (SIT) was superior to an attention control (AC) condition at improving psychological and relational well-being within a national sample of 349 custodial grandmothers (CGM). However, because the corresponding effect sizes (ES) were modest (range =.22 -.41) and effects waned over time, the present investigation used the same RCT data to investigate moderators of differential treatment efficacy for psychological and relational outcomes at both immediate (T1) and nine-months (T5) post intervention. Multiple potential moderators (including CGM adverse childhood experiences, key contextual/demographic factors, and baseline values of all study outcomes) were first entered simultaneously into classification and regression tree analyses to identify predictors of treatment efficacy within the SIT condition at T1 and T5. Less favorable baseline values of the study outcomes emerged as the only significant predictors of treatment-related change in the SIT condition, with generally large effect sizes (.50 - 1.44) observed. To establish whether these predictors were true moderators of differential treatment efficacy, a series of MANOVAs were then conducted to see if these predictors interacted significantly with RCT condition. At T1 and T5, moderation was scarce across psychological outcomes (i.e., depressive and anxious symptoms, self-esteem, and loneliness) but plentiful across relational outcomes (i.e., prosocial behavior, attachment with grandchild, and peer and grandchild relational quality). We conclude that, SIT is a promising intervention for enhancing the relational well-being of CGM, especially for those with poorer baseline levels. [Funded by R01AG054571]

